# Metabolic Surgery and Cancer Risk: An Opportunity for Mechanistic Research

**DOI:** 10.3390/cancers13133183

**Published:** 2021-06-25

**Authors:** Edward R. Sauter, Brandy Heckman-Stoddard

**Affiliations:** Division of Cancer Prevention, National Cancer Institute, Rockville, MD 20850, USA; brandy.heckman-stoddard@nih.gov

**Keywords:** metabolic surgery, bariatric surgery, cancer risk, cancer mortality

## Abstract

**Simple Summary:**

Metabolic (bariatric) surgery (MBS) provides the greatest maximum and sustained weight loss among individuals who are morbidly obese. It is more effective than lifestyle interventions in improving or eliminating type 2 diabetes mellitus (T2DM) and in decreasing cardiovascular (CV) risk. Preclinical studies have been conducted to investigate the mechanisms by which MBS leads to the benefits in T2DM and CV risk. In this review, we describe the emerging evidence that MBS may also impact cancer risk and mortality, and whom may benefit most. We describe the long term involvement and commitment of the National Institutes of Health in obesity research in general and MBS in particular. We outline the need for additional research to understand the mechanism(s) by which MBS may influence cancer, since these mechanism(s) are currently unknown.

**Abstract:**

Metabolic (bariatric) surgery (MBS) is recommended for individuals with a BMI > 40 kg/m^2^ or those with a BMI 35–40 kg/m^2^ who have one or more obesity related comorbidities. MBS leads to greater initial and sustained weight loss than nonsurgical weight loss approaches. MBS provides dramatic improvement in metabolic function, associated with a reduction in type 2 diabetes mellitus and cardiovascular risk. While the number of MBS procedures performed in the U.S. and worldwide continues to increase, they are still only performed on one percent of the affected population. MBS also appears to reduce the risk of certain obesity related cancers, although which cancers are favorably impacted vary by study, who benefits most is uncertain, and the mechanism(s) driving this risk reduction are mostly speculative. The goal of this manuscript is to highlight (1) emerging evidence that MBS influences cancer risk, and that the potential benefit appears to vary based on cancer, gender, surgical procedure, and likely other variables; (2) the role of the NIH in MBS research in T2DM and CV risk for many years, and more recently in cancer; and (3) the opportunity for research to understand the mechanism(s) by which MBS influences cancer. There is evidence that women benefit more from MBS than men, that MBS may actually increase the risk of colorectal cancer in both women and men, and there is speculation that the benefit in cancer risk reduction may vary according to which MBS procedure an individual undergoes. Herein, we review what is currently known, the historical role of government, especially the National Institutes of Health (NIH), in driving this research, and provide suggestions that we believe could lead to a better understanding of whether and how MBS impacts cancer risk, which cancers are impacted either favorably or unfavorably, the role of the NIH and other research agencies, and key questions to address that will help us to move the science forward.

## 1. Introduction

This text is not meant to be an exhaustive review of metabolic (bariatric) surgery (MBS) procedures, nor its impact on disease in animal or human studies. Rather, we focused on what we felt were key publications that moved the relevant field forward.

**Figure 1:**
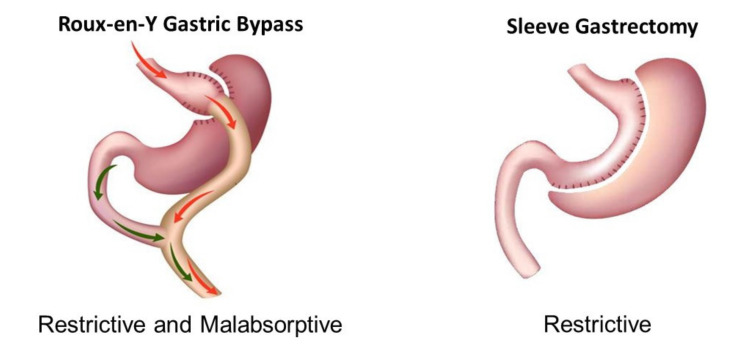
Common metabolic (bariatric) surgical procedures.

Metabolic (bariatric) surgery (MBS) has been performed since 1954. To decrease food absorption, early procedures bypassed a portion of the small intestine by connecting the upper to the lower intestine with various modifications to decrease morbidity from the procedure due to malabsorption [1]. The two most commonly performed MBS procedures today are the Roux-en-Y gastric bypass (RYGB) and the sleeve gastrectomy (SG), comprising 78% of all MBS procedures performed in the U.S. in 2018 [2]. The adjustable gastric band was commonly performed in the past, but not currently (Figure 1). The RYGB is thought to be both restrictive and malabsorptive as it decreases the amount of food that can be consumed (restrictive) and bypasses most of the stomach and duodenum (malabsorptive), whereas the SG is restrictive but not as malabsorptive since the procedure restricts the amount of food that can be consumed (restrictive) but does not bypass the stomach or intestine (Figure 1). Malabsorptive procedures generally alter bile acid concentrations in the intestine more than restrictive procedures. Most MBS procedures are now done laparoscopically. Laparoscopic MBS has a mortality rate of 0.1% and complication rate of 4%, which compares favorably with commonly performed surgical procedures such as gallbladder surgery, appendectomy, and knee replacement [3].

The average patient loses 70% of their excess body weight in the first 1–2 years after RYGB surgery [4]. The prospective Swedish Obese Subjects (SOS) study reported an average 32% total body weight loss 1–2 years after RYGB with sustained weight loss of 27% 15 years after surgery compared to 1% weight gain after the start of a lifestyle weight loss intervention [5]. In the same study, vertical banded gastroplasty (a restrictive procedure that was commonly performed in the past), demonstrated on average a total weight loss of 25% in the first 1–2 years with an average sustained loss of 18% at 15 years. Most MBS studies comparing RYGB to restrictive MBS procedures demonstrate greater weight loss after RYGB, with more side effects after RYGB due to the more complex nature of the surgical procedure and to postsurgical deficits in vitamin B12, iron, calcium, and folate.

There is evidence that MBS decreases type 2 diabetes mellitus (T2DM), cardiovascular (CV) risk, sleep apnea, and obesity driven cancers [5]. There is a significant improvement in glucose regulation among patients with T2DM who undergo MBS (improved insulin sensitivity, lower Hb1Ac) before significant weight loss [4]. The evidence of improvement in T2DM before much weight loss suggests that the health benefits of MBS are not simply due to the dramatic initial and sustained weight loss that occurs among most patients who undergo MBS. Using data from healthcare systems in the United Kingdom and Scandinavia, which provide some level of universal health care, MBS among eligible individuals was found to reduce healthcare costs, extend overall life on average by 0.8 years, and increase quality adjusted life by four years [6]. Nonetheless, MBS is only performed on one percent of the affected population [7]. There are clear changes in gut peptides and bile acids [4], adipose tissue function (with changes in adipokine production and decreased adipose tissue inflammation) [8], gut-brain signals (which lead to satiety despite decreased food consumption) [9], intestinal gluconeogenesis (an improvement in which has protective effects against diabetes and obesity by positively regulating glucose homeostasis and hepatic glucose production) [10], and an improved CV metabolic profile, including improvements in total cholesterol, triglycerides, and high and low density lipoproteins [11]. These observations provide convincing evidence that MBS consistently improves and often eliminates T2DM and lowers CV risk.

## 2. Animal Models of MBS

Both large and small animal models have been developed to study the effects of MBS [12]. Rodent animal models are most often used due to feasibility (low cost, ease of housing). Rodent models have extensively studied the mechanism(s) behind the metabolic improvements that occur after MBS [13] for the reduction or elimination of T2DM [14] and CV risk [15]. These studies have demonstrated significant changes after MBS in gut hormones [12], the increase after MBS in intestinal gluconeogenesis and its importance in weight loss and improved glucose control [13], how changes in gut microbiota and bile acid delivery after MBS improve T2DM [14], and how altered tissue sensitivity to ghrelin after MBS appears to decrease myocardial infarction size [15]. Studies also suggest that MBS alters gut-brain crosstalk [9] and may improve adipose tissue function [8].

On the other hand, there is a paucity of mechanistic animal studies evaluating the role of MBS in cancer, despite substantive epidemiologic evidence supporting the benefit of MBS in cancer. This is despite the fact that both rat and murine models of mammary and other cancers generally form within 6 months or less in animals that develop diet induced obesity and that on average live 2 or more years, making assessment of mechanistic changes that influence cancer feasible.

## 3. The National Institutes of Health (NIH) Focus on Obesity

The Strategic Plan for NIH Obesity Research was first published in March 2011 (https://www.obesityresearch.nih.gov/strategic-plan, accessed on 24 June 2021). In 2018–2019, the Obesity Research Task Force reaffirmed that the challenges and opportunities identified in the Plan reflect the current research landscape and should continue to guide obesity research. The first research opportunity outlined in the Strategic Plan was to “Discover Biologic Mechanisms Regulating Energy.” It is important to determine if the benefits of MBS are simply from weight loss, or if there are other mechanisms driving the benefit. The Strategic Plan points out that “MBS leads to resolution of diabetes before significant weight loss has occurred provides a model to explore potential mechanisms by which the gut communicates with the pancreas, liver, fat, and the central and peripheral nervous systems.” The amount of maximum and sustained weight loss varies by MBS procedure. The Strategic Plan proposed to “study the mechanisms by which different MBS procedures accomplish weight loss” using both humans and animals. “For example, study patients undergoing BS and animal models of MBS to expand the understanding of the role of gut signaling pathways and secreted molecules in regulating eating behavior, nutrient handling, and energy balance.”

### 3.1. Role of the NIH in MBS

The NIH has played an important role in MBS, not only in studying the science behind the procedure, but also in assessing its efficacy and safety, as well as developing recommendations through hosting three consensus conferences (Table 1). The purpose of the consensus conferences was to summarize the state of knowledge of MBS. Proceeding summaries and recommendations of the expert panel were published [16]. These summaries and recommendations were very important in the development of MBS.

Held in Bethesda, MD in 1978, the first conference was sponsored by the NIH’s National Institute of Arthritis, Metabolism, and Digestive Diseases (currently the National Institute of Diabetes, Digestive and Kidney Disease, NIDDK), to discuss the current status of surgical treatments for morbid obesity [17]. The stated goal was to obtain a consensus among knowledgeable experts in the field regarding surgical treatment(s) for MBS. A consensus was reached that MBS procedures should be confined to those with very severe, or morbid, obesity. An important part of the discussion was to define what constituted morbid obesity. It was decided that individuals who were 100 pounds or more overweight were morbidly obese, translating to a body mass index (BMI) of approximately 40 kg/m^2^ for an average adult male.

The first conference addressed the surgical treatment of morbid obesity with gastric or intestinal (jejunoileal) bypass operations. At this time, various gastric bypass procedures were performed in an attempt to identify which worked best. The report noted that MBS was effective in reducing weight but had a number of undesirable complications. The report highlighted that further study was needed into the mechanism(s) of the effects of the surgical therapies, including the changes that occur in gut hormones and their responses to food ingestion, the possible role of gut hormones as satiety signals and possible neural stimuli arising from the gastrointestinal tract.

A second NIH consensus conference on MBS was held in 1985 that focused on the health implications of obesity. It was established that obesity increased the risk of CV disease, dyslipidemia, diabetes mellitus, gallbladder disease, and increased the prevalence and mortality from cancer [18].

In 1991, a third consensus conference convened by NIDDK also focused on the health implications of obesity. A panel of experts reviewed published literature as well as oral presentations and responded to questions from the audience in order to construct a consensus statement. It was recognized that nonsurgical management of morbid obesity yields weight loss in some, but weight loss maintenance fails in most. Two MBS procedures were common at the time and were the focus of discussion: vertical banded gastroplasty and gastric bypass, especially RYGB. It was noted that the jejunoileal bypass was no longer commonly performed in the U.S. due to a higher frequency of metabolic complications. There was evidence reported that MBS procedures were associated with reductions in glucose intolerance, diabetes mellitus, hypertension, sleep apnea, and lipid abnormalities.

The panel made the following recommendations:

1. Patients seeking effective therapy for severe obesity for the first time should participate in a nonsurgical program, including a dietary regimen, appropriate exercise, and behavior modification. Transient success of very low-calorie diets, behavioral modification, and limited pharmacologic intervention was also noted.
2. Gastric restrictive or bypass procedures could be considered for well-informed and motivated patients with acceptable operative risks.
3. Patients who were candidates for surgical procedures should undergo evaluation by a multidisciplinary team.
4. The operation should be performed by an experienced surgeon in an appropriate clinical setting.
5. Lifelong medical surveillance after surgical therapy is a necessity.
6. Specific criteria for operative intervention were determined to be patients with a BMI > 40 kg/m^2^ as well as patients with a BMI 35–40 kg/m^2^ who have high risk comorbid conditions, such as cardiopulmonary disease, severe diabetes, and physical problems that interfered with their lifestyle (for example, employment, family functioning, and ambulation).

In 1995 the National Heart, Lung, and Blood Institute (NHLBI) and NIDDK convened an Expert Panel on the Identification, Evaluation, and Treatment of Overweight and Obesity in Adults to determine if the 1991 guidelines should be updated. The Panel performed a systematic review of the published scientific literature from 1980 to 1997 on topics identified by the panel as relevant to their task. Definitions of overweight (BMI = 25.0–29.9 kg/m^2^), obesity (BMI = 30–39.9 kg/m^2^), and morbid (a.k.a. severe or extreme) obesity (BMI ≥ 40 kg/m^2^) were confirmed. The expert panel found no reason to alter the 1991 consensus panel recommendations and confirmed the recommendation that MBS is an option for carefully selected patients with BMI ≥ 40 kg/m^2^ (There has some variability over time as to whether morbid obesity is a BMI ≥ 40 kg/m^2^ or > 40 kg/m^2^.) or 35 kg/m^2^ with comorbid conditions when less invasive methods of weight loss have failed and the patient is at high risk for obesity related morbidity or mortality [19].

In 2008, NHLBI convened an expert panel to update the existing clinical guidelines on the management of overweight/obesity. During a panel review, the Institute of Medicine issued two reports that established new “best practice” standards for generating systematic evidence reviews and developing clinical guidelines. This led NHLBI to limit their role to completing a systematic evidence review of the topic and collaborating with other organizations to prepare and issue clinical guidelines. In 2013, NHLBI published “Managing overweight and obesity in adults: systematic evidence review from the obesity expert panel, 2013”. This led to the “2013 American Heart Association, the American College of Cardiology and The Obesity Society (AHA/ACC/TOS) guideline for the management of overweight and obesity in adults” [20]. The panel concluded that there was strong evidence (category A) for physicians to recommend MBS to their patients who had a BMI > 40 kg/m^2^ or BMI > 35 kg/m^2^ with an obesity related comorbid condition who have failed nonsurgical approaches with or without pharmacotherapy.

To facilitate research in the field of MBS, NIDDK established the Longitudinal Assessment of Bariatric Surgery (LABS) consortium based on an NIDDK-sponsored Working Group on Research in Bariatric Surgery. The Working Group, which was convened in 2002, advised NIDDK to establish a consortium of centers that perform MBS and develop a database to collect information on clinically important predictors and outcomes that would benefit clinical research and the understanding of MBS and its sequelae [21]. After a competitive peer review, six Clinical Centers and one Data Coordinating Center were established in 2003. The LABS study was divided into three phases. The primary goal of phase 1 (LABS-1) was short term MBS safety. Researchers followed 4776 adult participants with an average BMI of 46.5 kg/m^2^ who underwent MBS between 2005 and 2007. Complications including death rates within 30 days of surgery were assessed. In LABS-2, scientists followed more than 2400 people who had MBS between 2006 and 2009, measuring changes in the patients’ clinical, metabolic, and psychosocial characteristics, and the patient’s use of health care services following surgery. Clinical scientists met with participants before, six and 12 months after surgery, then annually through 2015. LABS-3 looked more closely into the specific effects of MBS. Some LABS-1 and -2 participants were followed through two targeted studies, one regarding how MBS affected glucose levels among MBS participants with T2DM at baseline, and the second determined how MBS affected participant mental wellbeing and eating behaviors. The LABS database and a limited number of clinical specimens (serum, plasma, and deoxyribonucleic acid) are available from the NIDDK biorepository. A TEEN-LABS study was conducted in teenagers who underwent MBS between 2007 and 2012. Follow-up in the TEEN-LABS study is continuing.

In addition to LABS, there are many concluded and ongoing NIH funded research projects to address the safety and clinical impact of MBS, as well as the mechanism(s) by which it works. One notable project is the SOS, a long-term MBS cohort study that has provided important insights into MBS. Though conducted in Sweden and primarily funded with Swedish resources, NIH has assisted in research analyses of the effect of nutrient intake on weight loss and other outcomes. In short, there has been a long-standing research emphasis by the NIH on MBS and MBS mechanisms in T2DM and CV disease risk reduction. On the other hand, the NIH focus on MBS and cancer is less extensive. There is relatively little funded research describing the role of MBS in cancer risk, and even less on the mechanisms that may drive alterations in cancer risk. The focus on T2DM and CV disease is not surprising, as the clinical benefits with T2DM and CV disease occur relatively soon after MBS, whereas clinical evidence demonstrating a cancer benefit take much longer. There are many reasons for this increased emphasis on cancer, including the rising prevalence of obesity both in the U.S. and worldwide, as well as an increasing number of epidemiologic studies relating MBS to cancer risk reduction (or, at least for colorectal cancer, a possible increased risk, as outlined below).

### 3.2. National Cancer Institute (NCI) Emphasis on Obesity

Obesity is one of four scientific priorities that are highlighted in the fiscal year (FY) 2022 NCI Annual Plan and Budget Proposal (https://www.cancer.gov/research/annual-plan/scientific-topics/obesity, accessed on 24 June 2021). As the plan outlines, almost 40% of adults are obese, including 35% of cancer survivors. Obesity increases the risk of death from cancer by 52% among men and 62% among women. The report mentions BS as an approach to control obesity that leads to a reduced risk of breast, colon, endometrial, and pancreatic cancers compared with obese controls.

The plan points out the importance of “uncovering the biology at the intersection of obesity and cancer,” and that it will be important to develop interventions based on the molecular mechanisms that drive obesity related cancer, such as changes in metabolism, hormone signaling, inflammation, and microbiome function. Additionally, the plan calls for studies evaluating the impact on cancer recurrence of weight loss among obese cancer survivors.

The plan mentions that in addition to the difficulty in losing weight, perhaps the biggest challenge with lifestyle weight loss strategies is weight loss maintenance. MBS has consistently been shown to be the most effective strategy to maintain weight loss once achieved. For example, in the largest prospective study of MBS, the SOS, participants who underwent RYGB lost on average 32% of total body weight 1–2 years after surgery with a sustained weight loss of 27% 15 years after surgery compared to 1% weight gain 15 years after the start of a lifestyle weight loss intervention [5]. Indeed, ~90% of overweight and obese individuals who lose weight through lifestyle interventions regain all of the lost weight. The Look AHEAD trial, which compared an intense lifestyle intervention to usual medical care in highly motivated overweight or obese patients with T2DM found that participants regained about half of the weight lost, for an average weight loss of 4.7% at 8 years. The study was terminated early since it failed to achieve its primary endpoint, a reduction in the incidence of adverse CV events. The study also did not identify a significant difference between the two groups in total cancer incidence, incidence of non-obesity related cancers, or total cancer mortality. There was a nonsignificant trend toward a reduction in obesity related cancers (hazard ratio 0.84, 95% CI: 0.68–1.04).

## 4. Impact of MBS on Cancer Risk and Mortality

There have been a number of studies (Table 2) assessing the potential impact of MBS on cancer risk (cancer incidence and/or cancer mortality). Most report a reduction in the risk of one or more obesity related cancers, with some also demonstrating a reduction in all cancer mortality. A meta-analysis of 21 cohort studies involving 304,516 obese patients who underwent MBS and 8.5 million obese controls found a decreased risk after MBS both in cancer incidence (OR = 0.56) and mortality (OR = 0.56). In subgroup analysis, MBS was associated with a significant decrease in breast and endometrial cancer risk, but not with other cancer risk [22]. A retrospective cohort study involving 22,198 subjects who had MBS and 66,427 matched obese controls observed a lower risk (HR = 0.67) of developing any cancer during 3.5 years mean follow-up compared to matched patients with severe obesity who did not undergo MBS, and results were even stronger when the outcome was restricted to obesity related cancers (HR = 0.59). Among obesity related cancers, the risk of postmenopausal breast (HR = 0.58, *p* < 0.001), colon (HR = 0.59, *p* = 0.04), endometrial (HR = 0.50, *p* < 0.001), and pancreatic cancer (HR = 0.46, *p* = 0.04) were each significantly lower among those who had undergone MBS [23]. The SOS study reported, after a mean of 24 years (22 years in the control) follow-up, a 23% lower risk of all-cause mortality and a 23% lower risk of death from cancer in the MBS group [24].

There is, unfortunately, little evidence from these same studies documenting the mechanism(s) driving cancer risk reduction other than weight loss itself. This lack of mechanistic evidence is perhaps not surprising, given the long time it takes for cancer to form compared to T2DM or CV disease among individuals who are obese but clinically disease free. Evaluating the association of cancer with MBS requires longer follow-up with its attendant challenges regarding cost and loss of individuals during the lengthy follow-up. Additionally, sequential samples are ideally required both at baseline and sequentially after surgery in order to identify the biomarkers most predictive of cancer risk reduction benefit, which often were used for other important investigations such as T2DM or CV disease impact. There may be opportunities to use samples collected within studies that were performed for the purpose of examining the effect MBS on T2DM or CV disease to understand the impact on cancer risk if long term follow-up has been performed.

### 4.1. Gender May Influence MBS Benefit

Multiple studies report a greater cancer risk reduction among women than men who underwent MBS (Table 3). In the SOS study, the number of cancers that were identified in women after MBS was lower than in the control group (HR = 0.58, *p* = 0.0001), whereas there was no effect of MBS in men (HR = 0.97, *p* = 0.90) [25]. Men comprised only 29% of the population evaluated, limiting the detection of an effect from MBS in men, should it exist. Six observational studies (*n* = 51,740) comparing cancer incidence in obese patients who underwent MBS vs. controls demonstrated a RR among patients undergoing MBS of 0.55, with the risk benefit modified by gender (*p* = 0.021). The pooled RR in women was 0.68 and in men 0.99 for MBS vs. control [26]. Studies to address the influence of gender on MBS benefit should evaluate if clinical variables among men who undergo MBS (BMI, cancer risk, etc.) are similar to those among women who undergo MBS.

Epidemiologic studies suggest that the beneficial effect of weight loss on cancer risk is greater in women than in men [31,32]. If true, possible reasons why this would be true include: (1) 80% of all MBS performed in the U.S. occur in women, making an effect of MBS, should it exist, easier to detect [33]; and (2) the most consistent beneficial effects in women’s cancers are in postmenopausal breast and endometrium, suggesting that the downregulation of aromatase and subsequent estrogen production driving these cancers in obese subjects was downregulated with weight loss. Indeed, one study comparing obese patients who underwent MBS to those who did not observed a decreased risk of cancer in hormone regulated cancers in both men and women (postmenopausal breast-OR = 0.25, endometrium-OR = 0.21 and prostate-OR = 0.37) [34].

### 4.2. Does MBS Increase the Risk of CRC?

Multiple publications report an increase in CRC risk among patients undergoing MBS compared to controls (Table 3). When observed, the increase in CRC risk after MBS was evident among both women and men. Indeed, more often than not the risk was greater in women than men. The increased CRC risk was observed both in studies with an increased [27] and decreased [28,34] overall cancer risk after MBS. The increased odds ratio was greater after gastric bypass than after sleeve gastrectomy or gastric banding in at least one study [27]. Other studies report reduced CRC risk after MBS [23,29]. Speculation as to the mechanism(s) behind an increase in risk include a decreased risk of statin use after MBS [30]. However, this possible mechanism does not explain: (1) why some reports indicate a decreased CRC risk after MBS; and (2) the altered microbiome that is observed, especially after RYGB due to the altered intestinal bile acid composition of the colon and a shift from a protein degradation to a protein putrefaction phenotype [35].

### 4.3. Do Race and Ethnicity Influence the Effect of MBS on Cancer Risk?

Cancer incidence and survival rates vary by race and ethnicity, as does the prevalence of obesity. The few reports evaluating the potential benefit of MBS based on race and ethnicity have assessed T2DM, CV, sleep apnea and other endpoints, but not cancer. The impact of race and ethnicity on cancer endpoints, and the mechanism(s) involved, are understudied areas worthy of further investigation [46,47].

### 4.4. Does the Specific MBS Procedure Influence Benefit?

There has been speculation that the type of MBS procedure may influence the benefit (or harm) of MBS regarding cancer (Table 3). As mentioned earlier, RYGB is both a malabsorptive and restrictive procedure, whereas SG and gastric banding are primarily restrictive procedures. Multiple MBS studies report greater maximum and sustained weight loss with RYGB than other MBS procedures [48,49]. Among MBS participants, after a median follow-up time of 2.7 years, those who underwent RYGB had greater weight loss, a slightly higher T2DM remission rate, less T2DM relapse, and better long-term glycemic control compared with those who had SG [48]. Moreover, gastric bypass has been found to decrease the risk of hormone related cancers (breast, endometrium, and prostate) to a greater extent than restrictive MBS procedures [34].

In one study, gastric bypass, but not gastric banding or SG, was associated with an increased risk of CRC [34]. RYGB and jejuno-ileal bypass (which is also both a malabsorptive and restrictive procedure) both demonstrate increased colonic proliferation after MBS [50]. RYGB was also found to increase the expression of COX-1 (inflammatory) and COX-2 (tumorigenic) in colonic epithelium [50]. It has been speculated that this is due to the increased exposure of colonocytes after these procedures to luminal secondary bile acids [50]. Whether the increase in proliferation after bypass procedures would increase CRC risk is unknown. Moreover, it is unknown if primarily restrictive MBS procedures would also increase colonic proliferation.

## 5. Proposed Mechanisms Leading to Cancer Risk Reduction after MBS

Two recent publications evaluated circulating biomarkers (one study) or circulating and tissue biomarkers (second study) before and after bariatric surgery to determine if they were associated with cancer risk reduction. The first study was retrospective, the cancers were not adjudicated, and it evaluated a bariatric surgery procedure rarely performed today. In addition, males were not independently evaluated [51]. The second study was small, and the endpoint was endometrial proliferation, not cancer [36]. Despite these challenges, the first study found that decreases from baseline in serum levels of glucose, proinsulin, insulin, and leptin as well as increases in ghrelin were associated with a lower risk of cancer, while the second observed favorable changes in endometrial tissue after bariatric surgery, including a reversion from atypia to normal, alterations in estrogen and progesterone expression, and PI3K-AKT–mTOR signaling, as well as in circulating biomarkers associated with obesity related cancer.

Due to the paucity of mechanistic studies evaluating how MBS influences cancer risk, speculation as to possible mechanisms generally look to mechanistic studies linking obesity with an increase in cancer risk. Many studies have already been reported. An excellent detailed review of the mechanisms is suggested in [52]. The mechanisms by which MBS may influence cancer risk, in general, are based on the mechanisms that drive obesity related cancer. Many of these mechanisms are also linked to T2DM and CV risk (Table 3). These include increased insulin sensitivity [36,39], decreased insulin-like growth factor (IGF)-1 [37], altered intestinal bile acid concentration [45], decreased adipose tissue and systemic inflammation (with lower levels of circulating inflammatory cytokines) [36,52], a favorably altered gut microbiome [43,46] and epigenetic changes [42], decreased adipocyte size [41] (which allows more normal adipocyte functioning), decreased aromatase production [44] and enzymatic conversion of androgens to estrogen [36] thereby limiting the increase in circulating estrogen, lowering oxidative stress [38], and increasing intestinal gluconeogenesis [10,40]. Some of these proposed mechanisms are supported by MBS animal studies, as we outline in in Section 3. These favorable mechanistic changes lead to lower cell proliferation [36]. The lack of mechanistic studies of MBS and cancer, either in animals or humans, points to the importance of investigation to understand how MBS influences cancer, when MBS effects are favorable vs. not, and could help guide for whom MBS is most beneficial.

## 6. Conclusions

There is convincing evidence that MBS favorably impacts T2DM and CV risk, and there is also mounting evidence that it decreases the incidence and mortality from some (but perhaps not all) obesity-related cancers. There are many unanswered questions regarding how MBS influences cancer, including: (1) What are the mechanism(s), other than weight loss, that influence cancer risk (cancer development and/or cancer mortality); (2) Is MBS more beneficial in cancer risk reduction in women than men, and if so, why? (3) Does MBS increase or decrease the risk of CRC, and if so, what are the mechanism(s)? (4) Which cancers are favorably (or unfavorably) impacted by MBS, and why? and (5) Does the specific MBS procedure influence cancer impact?

The latest NCI plan and budget have a strong emphasis on the role of obesity in cancer, as well as strategies to mitigate the risk that obesity presents. It will be essential to understand the mechanisms driving obesity-related cancer, and to better understand the impact on cancer recurrence of weight loss among obese cancer survivors. To date, studies of behavior interventions have been able to achieve the desired weight loss in the short term but have failed to sustain the weight loss over the long term, resulting in a minimal effect on cancer outcomes. Due to its impact on sustained weight loss, MBS creates an opportunity to understand the impact of weight loss on cancer risk and mortality. Understanding the mechanism(s) involved has the potential to drive innovation to create targeted therapies to reduce the risk of obesity related cancer.

One of the challenges in addressing the mechanisms that drive the influence of MBS on cancer is the long time and large sample size required with human studies to observe an impact. There are funding challenges, recruitment and follow-up challenges, as well as biobank sample collection and storage challenges. There are hints from human MBS epidemiologic and biomarker studies at some of the mechanistic targets to further investigate [27]. Human studies are limited by sample size and their retrospective nature, where the effect of MBS on cancer was not part of the initial study design. These challenges are mitigated by performing animal studies of obesity, MBS and cancer. Rodent animal models are most often used due to feasibility (low cost, ease of housing). Rodent models have extensively studied the mechanism(s) behind the benefits of MBS for the reduction or elimination of T2DM and CV risk, with very few similar studies related to cancer. This is despite the fact that both rat and murine models of mammary and other cancers form within six months or less in animals that develop diet induced obesity and that the animals live on average for 2 or more years, making assessment of mechanistic changes that influence cancer feasible. There is an opportunity to understand the role of MBS in cancer risk through mechanistic studies, including the use of animal models, since a cancer endpoint is feasible in a two to five year timeline, whereas the timeline is much longer and more expensive for human studies. As we outline above, there are a limited number of human biomarker studies, which could provide hints at mechanistic targets to address, including changes in insulin resistance, chronic inflammation, gene pathway signaling, and hormone expression that appear to occur after MBS [27,36,51].

## Figures and Tables

**Table 1:** The role of NIH in MBS.

Title	Dates	Institute(s)	Purpose	Focus of Surgical Procedures Discussed	Outcomes
Consensus Development Conference on Surgical Treatment of Morbid Obesity	12.4–5.1978	NIDDK	Establish agreement among experts on use of emerging technologies for morbid obesity	GB, JI bypass	1. Improvement in CV and T2DM with weight loss after BS; 2. GB has fewer side effects than JI bypass; 3. surgery should be limited to patients with morbid obesity; 4. the mechanism(s) of the effect of MBS needs more attention
Consensus Conference on the Health Implications of Obesity	2.11–13.1985	NIDDK, NHLBI	What is obesity; what is the evidence that obesity adversely affects health; for what medical indications can weight loss be recommended; what is the evidence that obesity affects longevity; what should be the directions of future research in the area	Primary conference focus not on BS	Increased risk of CV disease, dyslipidemia, T2DM, gallbladder disease, increased prevalence of and mortality from cancer
Gastrointestinal surgery for severe obesity. Proceedings of an NIH Consensus Development Conference	3.25–27.1991	NIDDK	Assess the MBS treatments for obesity, the criteria for selection of MBS, the efficacy and risks of MBS, and the need for further research on MBS therapies	VBG, RYGB	(1) MBS restrictive or bypass (malabsorptive) procedures could be considered for candidates who are good surgical risks; (2) MBS surgical candidates should be evaluated by multiple disciplines with medical, surgical, psychiatric and nutritional expertise; (3) the MBS procedure should be performed in a setting with adequate support for all aspects of MBS care; (4) specific BMI criteria for surgery; (5) lifelong medical surveillance after MBS in needed
Expert Panel on the Identification, Evaluation, and Treatment of Overweight and Obesity in Adults	Panel convened May 1995	NHLBI, NIDDK	To evaluate published randomized clinical trials published between 1980 and 1997 to determine optimal treatment for overweight and obesity	VBG, RYGB	MBS is an option for subjects with BMI > 40 or > 35 kg/m^2^ with comorbid conditions when medical management has failed
Managing Overweight and Obesity in Adults: Systematic Evidence Review from the Obesity Expert Panel	2008	NHLBI	Expert panels were convened to update the existing clinical guidelines on cholesterol, blood pressure, and overweight/obesity, by conducting rigorous systematic evidence reviews.	VBG, adjustable gastric banding, RYGB, SG	MBS produces greater initial and maintained weight loss than other weight loss approaches. In obese adults, MBS generally results in a more favorable impact on obesity-related comorbid conditions than that produced by usual care, conventional medical treatment, lifestyle intervention, or medically supervised weight loss. MBS results in greater improvement in obesity-related comorbid conditions than nonsurgical approaches. Complications, weight loss and remission-related comorbidities following MBS vary by procedure.

NIDDK = National Institute of Diabetes, and Digestive and Kidney Disease; NHLBI = National Institute of Heart Lung and Blood Institute; RYGB = Roux-en-y gastric bypass; VBG = vertical banded gastroplasty; SG = sleeve gastrectomy; BPD = biliopancreatic diversion; MBS = metabolic (bariatric) surgery; T2DM = type 2 diabetes mellitus.

**Table 2:** Effects of MBS on cancer incidence and mortality.

Study Design	Study Groups/Numbers	Treatment Procedure(s)	Cancer Incidence +/- Mortality	Cancer Effect				Reference
				Overall	Cancer	Females	Males	
Prospective matched cohort	T: 2010; C: 2037; F: 2867; M: 1180	GB, VBG, RYGB	Cancer incidence, mortality	Incidence HR = 0.67 (*p* < 0.01); mortality HR = 0.77, *p* < 0.01	Melanoma HR = ND, *p* < 0.01; Hematologic HR = 0.16, *p* = 0.015; Other cancers HR = 0.40; *p* = 0.04	GB HR = 0.54, *p* = 0.026; VBG = 0.60, *p* < 0.01; RYGB HR = 0.54, *p* = 0.11	HR = 0.97, NS	[25,26]
Retrospective matched cohort	T: 22,198; C: 66,427; F: >80%	Various MBS procedures	Cancer incidence	HR = 0.67, *p* < 0.01; obesity related cancers HR: 0.59, *p* < 0.01	Postmenopausal breast HR = 0.58, *p* < 0.01; colon HR = 0.59; *p* = 0.04; endometrial HR = 0.50, *p* < 0.01; pancreatic cancer HR = 0.46, *p* = 0.04	HR = 0.64, *p* < 0.01), obesity associated cancers (HR = 0.58, *p* < 0.01), not obesity associated (HR 0.74, *p* < 0.01)	No significant cancer reduction for any cancer type	[24]
Meta analysis	T: 304,516; C: 8,492,408; both M/F	Various MBS procedures	Cancer incidence, mortality	Incidence OR = 0.56, *p* < 0.01; mortality OR = 0.56, *p* < 0.01	Breast HR = 0.49, *p* < 0.01; CRC 0.82, *p*,0.01; Endometrial HR = 0.43, *p* = 0.01	Not studied		[23]
Retrospective matched cohort	T: 8794; C: 8794	Bypass (including RYGB), GB, SG	Cancer incidence	Focus on obesity related cancers	Hormone related cancers OR = 0.23 (RYGB OR = 0.16), breast OR = 0.25; endometrium OR:0.21, prostate OR:0.37; CRC OR = 2.63 (RYGB)	Hormone related cancers OR = 0.22; CRC OR = 3.01 (RYGB)	Hormone related cancers OR = 0.37; CRC OR = 2.01 (RYGB)	[27]
Retrospective observational cohort	T: 39,747 (F: 76.6%); C: 962,860 (F: 62.9%)	52% restrictive, 48% restrictive & malabsorptive	Cancer incidence	Focus on obesity related cancers	Breast SIR = 0.76; Uterus SIR = 2.98; Kidney SIR = 3.06; Lung SIR = 0.70; CRC SIR = 1.26; restrictive CRC SIR = 1.41; restrictive & malabsorptive CRC SIR = 1.05	CRC SIR = 1.19	CRC SIR = 1.41	[28]
Retrospective observational cohort	T: 13,123 (F: 77%); C: Swedish national registry	GB, VBG, Bypass (including RYGB)	Cancer incidence	Focus on obesity related cancers	Obesity related cancers SIR = 1.04; CRC SIR = 1.52; Kidney SIR = 2.68; Bypass SIR = 1.01; VBG SIR = 1.05; GB SIR = 1.05	Breast SIR = 0.54; Endometrial SIR = 2.15; CRC SIR = 1.28	Prostate SIR = 0.84; CRC SIR = 2.34	[29]
Retrospective observational cohort	T: 74,131(F: 77.9%); C: 971,217(F: 49.4%)	GB, SG, Bypass (including RYGB)	Cancer incidence	Focus on CRC	CRC SIR = 0.75	CRC SIR = 0.79	CRC SIR = 0.77	[30]

MBS: metabolic (bariatric) surgery; T: treated with MBS; C: control; F: females; M: males; GB: gastric banding; VBG: vertical banded gastroplasty; SG: sleeve gastrectomy; RYGB: Roux-en-y gastric bypass; CRC: colorectal cancer; NS: not significant; HR: hazard ratio; OR: odds ratio; SIR: standardized incidence ratio.

**Table 3:** Mechanisms possibly linked to cancer risk reduction or increase.

Proposed Mechanism	How the Mechanism Influences Obesity Related Cancer	Reference
↓ Cell Proliferation	Lower cell proliferation→lower cell division→lower mutation rate	[36]
↓ Chronic Inflammation	↓ growth factors and cytokines→↓cell proliferation, cell survival and migration	[36,37]
↓ Oxidative Stress	↓chronic inflammation→↓oxidative stress→↑antioxidant enzymes, ↓OS byproducts, ↓ cancer risk	[38]
↓ Insulin Resistance	Decreased cell proliferation and increased apoptosis	[36,39]
↑ IGN	IGN improves insulin sensitivity, energy homoeostasis and exerts anti-obesity effects	[10,40]
↓ IGF-1	↓growth factor expression→↓cell proliferation, cell survival and migration↓	[37]
↓ Adipocyte Size	Normalizes adipocyte function	[41]
Epigenetic ∆ in Adipocytes	Favorable changes in obesity epigenome after MBS	[42]
∆ Adipokine Expression	↓ growth-promoting adipokines, ↑ adiponectin decreases IGF-1	[41]
∆ Gut Microbiome	Gut microbiota types influence obesity; levels change after bariatric surgery	[14,43]
↓ Aromatase	Fewer adipocytes→lower aromatase levels Lower hormone receptor levels leads to lower cell proliferation from hormone signaling	[44]
↓ ER, PR ↑ Bile Acid Concentration ∆ Concentrations of Gut Hormones	May increase colonic proliferation Influences brain-gut crosstalk	[45] [9]

IGN = intestinal gluconeogenesis; IGF = insulin-like growth factor; MBS = metabolic(bariatric) surgery; ER, PR = estrogen receptor, progesterone receptor.
